# Determination of Polycyclic Aromatic Hydrocarbons in Sludge from Water and Wastewater Treatment Plants by GC-MS

**DOI:** 10.3390/ijerph16142604

**Published:** 2019-07-22

**Authors:** Chih-Feng Chen, Yun-Ru Ju, Yee Cheng Lim, Shu-Ling Hsieh, Mei-Ling Tsai, Pei-Pei Sun, Ravi Katiyar, Chiu-Wen Chen, Cheng-Di Dong

**Affiliations:** 1 Department of Marine Environmental Engineering, National Kaohsiung University of Science and Technology, Kaohsiung 81157, Taiwan; 2 Department of Seafood Science, National Kaohsiung University of Science and Technology, Kaohsiung 81157, Taiwan

**Keywords:** PAHs, GC-MS, sludge, drinking water treatment plants (DWTP), wastewater treatment plants (WWTP)

## Abstract

The qualitative and quantitative analysis of 16 polycyclic aromatic hydrocarbons (PAHs) in sludge samples from drinking water treatment plants (DWTP) and wastewater treatment plants (WWTP) were established using gas chromatography–mass spectrometry (GC-MS). The method was suitable to quantify PAHs in the sludge of DWTP and WWTP and it was confirmed by the relevant quality assurance/quality control (QA/QC) procedures. The recovery of individual PAHs in the spiked samples ranged from 74.3% to 108.7%. Detection limits of the analytical procedure were 0.0010–0.0046 mg/kg dw for individual PAHs. This method was used to determine the concentration of PAHs in the selected two DWTP and four WWTP sludge samples. The results showed that the total PAHs (∑PAHs) were in low levels which ranged from 0.0668 to 0.1357 mg/kg dw, and 0.5342–1.0666 mg/kg dw for DWTP and WWTP respectively. The 3- & 4-ring PAHs were predominant in DWTP sludge, ranging from 77.4% to 82.7%; the 4-ring PAHs were predominant in WWTP sludge, ranging from 40.7% to 47.6%. The PAHs of DWTP sludge are mainly composed of 3-ring phenanthrene and anthracene and 4-ring pyrene, and chrysene. The PAHs of WWTP sludge are dominated by 4-ring fluoranthene, pyrene, and chrysene. The detected PAHs concentration should be undoubtedly considered for agriculture in sludge applications based on the limits of the EU regulations. The results of this study can be used for regular monitoring to establish a reference for sludge management and application to agriculture.

## 1. Introduction

With the growth of urbanization, changes in industrial structure and raising awareness of environmental protection, drinking water treatment plants (DWTP) and wastewater treatment plants (WWTP) have been extensively built. The quality of drinking water and rates of sewage treatment has also increased. Although DWTP and WWTP ensure the safety of drinking water and reduce environmental water pollution problems, the sludge they produce is a potential threat to the environment. The sludge may contain hundreds of organic toxic compounds (e.g., polycyclic aromatic hydrocarbons (PAHs), phthalate esters (PAEs), alkylphenol polyethoxylates, synthetic musks, antibiotics, ultraviolet stabilizers, bisphenol analogs, organochlorine pesticides, polybrominated diphenyl ethers (PBDE), pharmaceuticals, hormones, perfluorinated compounds, and polychlorinated biphenyls (PCB)), heavy metals (Pb, Cr, Cu, Ni, Hg, and Cd), and pathogenic bacteria [1,2,3,4]. Improper disposal of sludge may cause secondary pollution to soil, groundwater, surface water and air [4].

PAHs are the hydrophobic organic compounds and tend to be bioaccumulated in the organisms [5,6]. Some PAHs have been identified as carcinogenic, mutagenic, and classified as a priority pollutant by the US Environmental Protection Agency (US EPA) and the European Union (EU). Therefore, PAHs are one of the most common target compounds in sludge-related research, especially the priority 16 PAHs [3]. Stevens et al. [1] surveyed the sludge collected from 14 wastewater treatment plants in the UK and found they contained a total of 24 PAHs concentration of 67–370 mg/kg dw. A review that contains the peer-reviewed literature and official government reports in the US indicated PAHs concentration in sludge ranged from below the detection limit to 199 mg/kg dw [2]. PAHs were one group of the most commonly studied organic compounds in sludge in China. Meng et al. [3] compiled a review and reported that the PAHs concentration of 0.1–170 mg/kg dw in sludge was measured from previous studies that were published during 1999–2012. The variation in PAHs concentration in sludge is considerable, mainly depending on the nature of the wastewater, treatment plant procedures, and geographical differences [7]. The final processing methods of sludge mainly include land application, incineration, and landfill, indicating that PAHs can re-enter the environment through air, water, and soil [3,8]. Therefore, before the final processing of sludge, the concentration level and risk information of pollutants such as PAHs are quite important for evaluating the subsequent fate and impact of specific compounds in the environment.

In Taiwan, about 2.66 million tons of WWTP sludge and 200,000 tons of DWTP sludge are produced each year [9,10]. At present, the final disposal methods of sludge are mainly landfill, incineration, heat treatment, and fertilizer. However, for the specification of various disposal methods, only the basic characteristic and heavy metals of sludge are regulated [11], and the criterion and information on organic pollutants are quite lacking. The purpose of this work was to analyze the PAHs in the sludge by using gas chromatography with mass spectrometer (GC-MS) and to confirm the applicability of analytical procedure by the relevant quality assurance/quality control procedures. In addition, this method was used to determine the concentration of 16 PAHs in the sludge from two DWTP and four WWTP in southwest Taiwan, and to evaluate the PAHs level, composition and potential toxicity in the sludge. The analytical procedures established in this study can be used as routine monitoring of DWTP or/and WWTP sludge to provide important information for sludge management and application strategies.

## 2. Materials and Methods 

### 2.1. Reagents and Standards

Chromatographic (HPLC) grade *n*-hexane and acetone were purchased from Echo Chemical Co. Ltd. (Miaoli, Taiwan). Analytical-grade anhydrous sodium sulphate (10–60 mesh) was from Avantor (Center Valley, PA, USA). The Copper powder was supplied by Sigma-Aldrich (Darmstadt, Germany) and washed with dilute nitric acid, reagent water, and acetone, and blown dry with nitrogen before the analysis. Standards of 16 PAHs in a 1000 mg/L mixture solution, deuterated PAHs internal standard solutions (naphthalene-d_8_, acenaphthene-d_10_, phenanthrene-d_10_, chrysene-d_12_, and perylene-d_12_) at 2000 mg/L, and surrogate standard solutions (2-fluorobiphenyl and 4-terphenyl-d_14_) at 1000 mg/L were obtained from AccuStandard Chem. Co. (New Haven, CT, USA). The standard working solutions of PAHs mixture, internal standard mixture and surrogate standard mixture were properly diluted with HPLC grade n-hexane and prepared freshly before the analysis. All glassware was rinsed with *n*-hexane and dried in an oven at 105 °C. Other materials were previously washed with ultrapure water and acetone.

### 2.2. Sampling

Dewatered sludge samples from two DWTP and four WWTP located in southwestern Taiwan were collected in November 2018. The two selected DWTP (DW1 and DW2) water sources are rivers and reservoirs, respectively; the selected four WWTP (WW1–WW4) inflow raw water is domestic sewage, and the WW2 sewage plant inflow raw water includes domestic sewage and intercepted water from polluted canals (volume ratio 1:2). The collected DWTP sludge sample is subjected to concentration and dehydration procedures, and the WWTP sludge sample is subjected to a concentration, digestion and dehydration process. Among them, WW1 and WW3 sludge are aerobic digestion, while WW2 and WW4 sludge are anaerobic digestion. About 1 kg of sludge was collected in a brown glass container previously washed with *n*-hexane and transported directly back to the laboratory. In the laboratory, the samples were freeze-dried for 72 h, ground to pass through a 1.0 mm sieve and fully homogenized. The dried sludge was placed in −20 °C in amber glass bottles pre-washed with *n*-hexane and covered with solvent-rinsed aluminum foil until further processing and analysis [12].

### 2.3. Sample Preparation

The sludge samples were extracted according to the method of Dong et al. [13] with slight modifications. Briefly, one g of dry and homogenized sludge sample was put into a clean glass test tube, and a 5 mL acetone/*n*-hexane (1:1), and 10 μL surrogate standard mixture solutions (20 mg/L) were then added. The sample tubes were mixed using the vortex (1 min) and extracted by the ultrasonic treatment (15 min). Mixed sludge and organic phase were separated by centrifugation at 3000 rpm for 10 min. The organic layer containing the extracted compound were collected into another clean glass tube using a Pasteur pipette, and the residual sludge was re-extracted twice with 1:1 (v/v) acetone/*n*-hexane (5 mL). All extracts were combined, and activated copper was added to the extract for desulphurization. Then, drying over anhydrous sodium sulphate, and concentration to 0.5 mL using a gentle stream of nitrogen. An internal standard mixture solution (200 ng) was added to the extract to be analyzed using GC-MS. 

### 2.4. GC-MS Analysis

A GC-MS system that connects an Agilent 7890B GC (Agilent Technologies, Santa Clara, CA, USA) to an Agilent 5977A mass selective detector (MSD) and equipped with an Agilent 7693A autosampler (Agilent Technologies, Santa Clara, CA, USA) was used to analyse the PAH compounds. The separation column used is a 30 m, 0.25 mm i.d. HP-5MS capillary column (Hewlett-Packard, Palo Alto, CA, USA) coated with 5% phenyl-methylsiloxane (0.25 μm film thickness). The analytical parameter settings for the GC-MS system are listed in Table 1, and the ion mass program used for quantification is detailed in Table 2. Identity of PAHs in the samples was confirmed by the retention time within ±0.06 relative retention time (RRT) units of the RRT of the standard component and the relative intensities of confirmation ions within ±30% of the authentic PAHs standards. Sixteen PAHs were quantified using the response factors related to the respective internal standards based on a five-point calibration curve for individual compounds. In this study, the concentrations of PAHs were expressed on a dry-weight (dw) basis.

### 2.5. Quality Control

To ensure the accuracy and precision of the PAHs analysis process of this study, a five-point calibration standards (0.1 to 2.0 ng/μL) in solution, detection limits, procedural blank, check standard, sample duplicates, and matrix spike standards were carried out. One µL of each calibration standard (containing internal standards) was analysed, and the area of the primary characteristic ion (as indicated in Table 2) was tabulated against concentration for each target analyte. The internal standard method was used to quantify PAHs, calculating response factors (RFs) for each target analyte relative to one of the internal standards and obtaining the relative standard deviation (RSD) of RF. The RSD of RF for each analyte should be less than <20%. The detection limits were estimated from three times standard deviation from repeated (*n* = 7) analysis of 16 PAHs with a low concentration of 0.01 ng/μL and converted by the concentration factor and sampled mass.

To prevent the contamination during the analyzed procedure, procedural blank samples (*n* = 3), adding no sludge sample, were prepared by the same procedure from the extraction to the PAHs analysis. The standards used for quality control were prepared by adding the standard solution to 1:1 (v/v) acetone/n-hexane. This study selected sludge samples of DW1 and WW1 for matrix spike standards. The original PAHs concentrations in DW1 and WW1 respectively ranged from 0.0007–0.0119 mg/kg dw and 0.0064–0.1516 mg/kg dw. Then DW1 and WW1 were respectively spiked 50 μL (50 ng) and 200 μL (200 ng) of 1 ng/μL 16 PAHs mix standards. Therefore, the final PAHs concentrations in spiked DW1 and WW1 were respectively in 0.0507–0.0619 mg/kg dw and 0.0264–0.1716 mg/kg dw. The recovery of the spiked samples was determined by the measured concentration dividing by the final concentration of the sample after the addition. The aforementioned procedural blank, check standard, sample duplicates, and matrix spike standard were carried out for every 10 samples.

## 3. Results

### 3.1. GC-MS Separation and Identification

**Figure 1:**
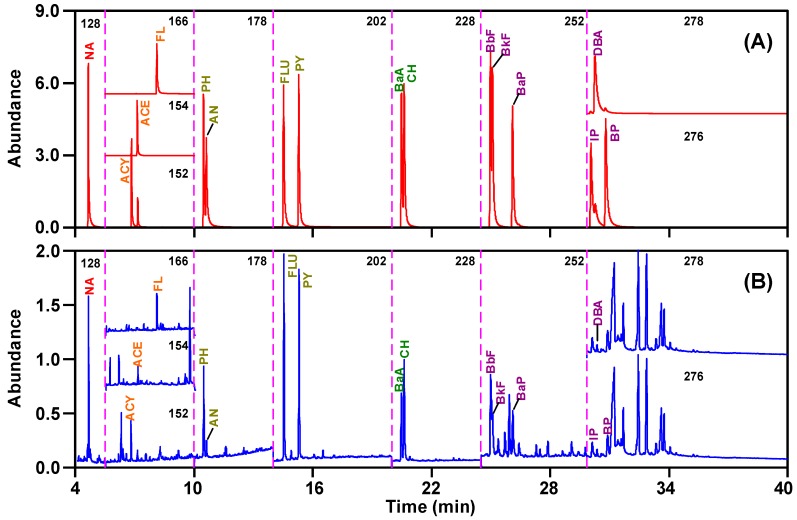
GC-MS selected quantification ion chromatograms of 16 PAHs in (**A**) standard mixture of 16 PAHs and (**B**) the WW2 sludge sample. The definitions of compound abbreviation see Table 2.

According to the set GC-MS conditions of Table 1 and Table 2, mixed standards of 16 PAHs were analyzed. The results showed that 16 PAHs could be effectively separated (Figure 1A). IS1 of internal standards was used for quantifying naphthalene, IS2 for acenaphthylene, acenaphthene, and fluorine, IS3 for phenanthrene, anthracene, and fluoranthene, IS4 for pyrene, benzo[a]anthracene, and chrysene, and IS5 for benzo[b]fluoranthene, benzo[k]fluoranthene, benzo[a]pyrene, indeno[1,2,3-cd]pyrene, dibenz[a,h]anthracene, and benzo[g,h,i]perylene. The separation and quantitation of PAHs in the sludge samples were achieved using the same GC-MS conditions as the standards. The 16 PAHs in the sludge samples were defined by the retention time and abundance of quantification/confirmation ions in the 16 PAHs standards. The selected quantification ion chromatograms of 16 PAHs in a standard mixture of 16 PAHs and the WW2 sludge sample was shown in Figure 1A,B. The peaks of 16 PAHs in the WW2 sludge sample were clearly defined and were not disturbed by the peaks of other organic compounds in the sludge (Figure 1 B). Therefore, the 16 PAHs of sludge samples can be quantified using the response factors related to the respective internal standards based on a five-point calibration curve for individual compounds.

### 3.2. Analytical Characteristics

The response factors based on the five-point calibration curve for individual compounds showed an acceptable RSD of 1.5 to 9.4%, the procedural blank values were always less than the detection limit, the recoveries of individual PAHs in check standards ranged from 86 ± 0.6% to 102 ± 2.1% (*n* = 3) and the relative percent differences of sample duplicates ranged from 1.7 ± 0.9% to 9.4 ± 3.6% (*n* = 6) for all of the target analyses (Table 3). The surrogate standard recoveries were 93.6 ± 7.1% for 2-fluorobiphenyl and 91 ± 9.3% for 4-terphenyl-d_14_ with sediment samples (*n* = 12). In addition, this study performed a matrix spike standard analysis to confirm the presence or absence of matrix interference in the sample and the appropriate analytical method. The recovery of each PAHs in the spiked samples ranged from 74.3 ± 2.3% to 108.7 ± 2.9% (*n* = 3), indicating that the analytical method of this study is suitable for the analysis of PAHs in sludge samples. The detection limits were 0.0010–0.0046 mg/kg dry weight for individual PAHs (Table 3).

### 3.3. Concentration Level of PAHs in Sludge

**Figure 2:**
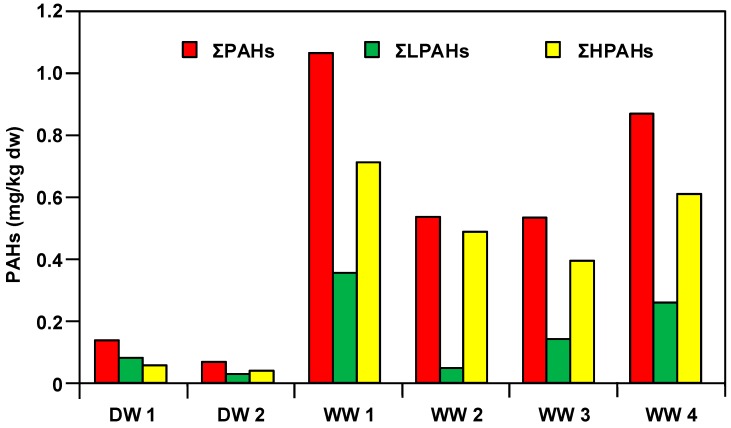
Distributions of ΣPAHs, ΣLPAHs, and ΣHPAHs in sludge samples of selected DWTP (DW1–DW2) and WWTP (WW1–WW4).

The distribution of 16 PAHs in sludge from selected DWTP (DW1 and DW2) and WWTP (WW1–WW4) is shown in Table 4. The concentrations of ΣPAHs in the WWTP sludge ranged from 0.5332–1.0666 μg/kg dw, which was 4–16 times higher than the DWTP sludge (0.0668–0.1357 mg/kg dw). This result shows that the PAHs of the artificially produced sewage are about one order of magnitude higher than the environmental water. The concentrations of ΣLPAHs (sum of 2- & 3-ring PAHs) and ΣHPAHs (sum of 4-, 5-, and 6-ring PAHs) in DWTP sludge were 0.0279–0.0793 mg/kg dw and 0.0389–0.0564 mg/kg dw, respectively; the concentrations of ΣLPAHs and ΣHPAHs in WWTP sludge were 0.0480–0.3550 mg/kg dw and 0.3931–0.7116 mg/kg dw, respectively. The ΣLPAHs/ΣHPAHs ratio is inconsistent in the two DWTP sludge, DW1 (ΣLPAHs/ΣHPAHs = 1.4) is greater than 1 and DW2 (ΣLPAHs/ΣHPAHs = 0.7) is less than 1. The ratios of ΣLPAHs/ΣHPAHs are consistently less than 1 (ΣLPAHs/ΣHPAHs = 0.1–0.5) in the four WWTP sludge, i.e., ΣHPAHs are significantly higher than ΣLPAHs (Figure 2). The concentration of ΣCPAHs (sum of 7 carcinogenic PAHs) varied in the range of 0.2101–0.3716 mg/kg dw in WWTP sludge, which is significantly higher than that in the DWTP sludge (0.0177 and 0.0409 mg/kg dw) (Table 4). The concentration of ΣCPAHs accounts for about 34.6–45.1% of PAHs in WWTP sludge, which is slightly higher than 26.5–30.1% of DWTP sludge. However, the ΣTEQ (the sum of BaP toxic equivalence quotient of carcinogenic PAHs, benzo[a]anthracene, chrysene, benzo[b]fluoranthene, benzo[k]fluoranthene, benzo[a]pyrene, indeno[1,2,3-cd]pyrene, and dibenz[a,h]anthracene [14,15]) of WWTP sludge (WW1–WW4) is between 0.0210–0.0372 mg/kg TEQ/kg dw, which is about 5–40 times higher than DWTP sludge (DW1: 0.0041 mg TEQ/kg dw and DW2: 0.0018 mg TEQ/kg dw).

**Figure 3:**
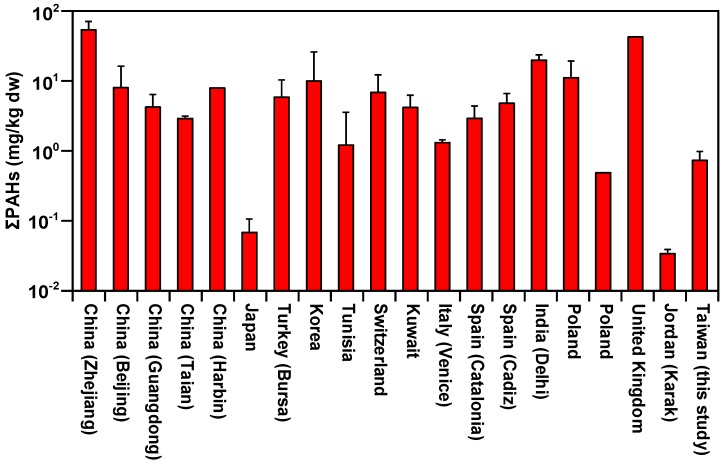
The mean concentrations and standard deviation of ΣPAHs in sludge from other studies around the world (Data list in Table 5).

Table 5 shows the comparison of the PAHs concentrations in sludge in this study with that from 19 other studies from around the world. For consistency, only the US EPA priority pollutants of 16 PAHs were selected to estimate ΣPAHs and ΣCPAHs in the 19 studies. The ΣPAHs content (0.53–1.07 mg/kg dw) of WWTP sludge in this study is higher than that of Poland (0.498 mg/kg dw) [16], Jordan (0.034 ± 0.005 mg/kg dw) [17], and Japan (0.069 ± 0.038 mg/kg dw) [18], similar to Italy (1.35 ± 0.13 mg/kg dw) [19] and Tunisia (1.25 ± 2.45 mg/kg dw) [20], but lower than most other countries’ WWTP sludge (Figure 3). The ΣCPAHs accounts for an average of 37% of ΣPAHs and falls within 12–74% of other studies.

In addition, since land application is one of the major ways for sludge disposal in countries around the world [21]. This study compares the limits of the relevant sludge applied to agricultural soils in the EU and China, due to the lack of relevant PAHs standards in Taiwan. In China, the PAHs contents allowed in sludge used for agriculture are benzo[a]pyrene 2 mg/kg dw and ΣPAHs 5 mg/kg dw [22]. The EU regulations for the use of sludge in agricultural soils are the sum of 9 PAHs (acenaphthene, phenanthrene, fluorene, fluoranthene, pyrene, benzo[b+j+k]fluoranthene, benzo[a]pyrene, benzo[g,h,i]perylene, and indeno[1,2,3-cd]pyrene) which is less than 6 mg/kg dw [23]. In this study, the PAHs of the DWTP and WWTP sludge did not exceed the Chinese standard benzo[a]pyrene 2 mg/kg dw and ΣPAHs 5 mg/kg dw as well as less than the EU limit 6 mg/kg dw of sum of 9 PAHs. This shows that based on the concentration of PAHs, the sludge in this study may be suitable for agricultural applications.

### 3.4. Composition of PAHs in Sludge

**Figure 4:**
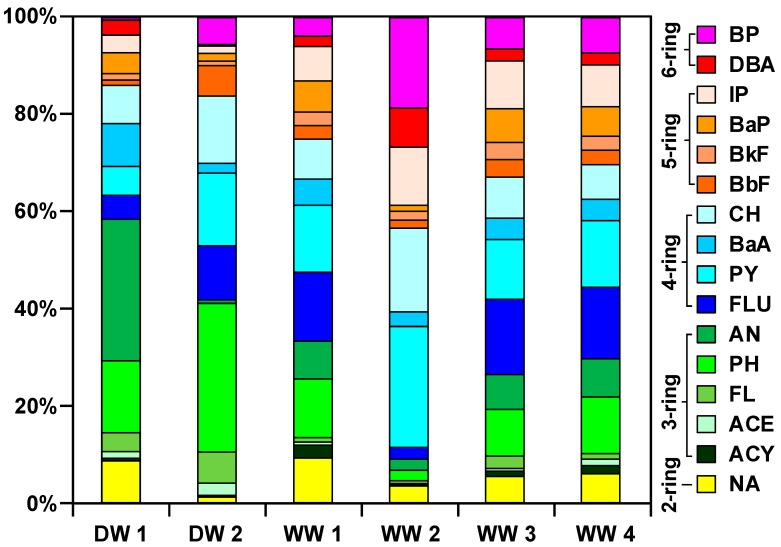
PAHs composition in sludge samples of selected DWTP (DW1–DW2) and WWTP (WW1–WW4). The definitions of compound abbreviation see Table 2.

The percentage contribution of 16 PAHs in the two DWTP and four WWTP sludge samples studied is shown in Figure 4. The highest content in DW1 sludge was observed for anthracene (29.2%) and phenanthrene (14.8%), while DW2 sludge was phenanthrene (30.6%), anthracene (14.9%), and chrysene (13.9%). This difference may be due to the fact that the raw water of the two DWTP is river water and reservoir water. The composition of PAHs in sludge may also vary due to the different organic composition of different water sources [7]. In WW1, WW3 and WW4 sludge, phenanthrene (9.6–12.1%), fluoranthene (14.2–15.5%), and pyrene (12.3–13.8%) are the most dominant, which is consistent with the results of previous studies on PAHs composition of municipal wastewater treatment plant sludge [27,37]. The WW2 sludge has the highest content of pyrene (24.9%), benzo[g,h,i]perylene (18.6%), and chrysene (17.3%), which is different from the other three types of sludge studied (Figure 4). Among the four WWTP in this study, the influent water of WW1, WW3, and WW4 is mainly domestic sewage, while the influent water of WW2 includes domestic sewage and polluted river water intercepting the Tainan Canal, and its water is subjected to industrial wastewater, surface runoff, and domestic sewage from the river bank. This may be the reason why the PAHs composition of the WW2 sludge is different from other sludge. Since pyrene and benzo[g,h,i]perylene are designated as traffic-related, and benzo[g,h,i]perylene is identified as a tracer of auto emissions [38,39,40], and chrysene is suggested to indicate industrial waste incinerators [40,41], therefore WW2 sludge has a high proportion of pyrene, benzo[g,h,i]perylene, and chrysene should be affected by surface runoff and industrial wastewater.

According to the number of aromatic rings, the 16 PAHs were divided into 2 to 6 ring PAHs [42]. The 3- & 4-ring PAHs were predominant in DW1 and DW2 sludge samples, ranging from 77.4% to 82.7% (Figure 4); the percentage compositions are 1.2–8.6% and 14–16.2% for the 2-ring and 5- & 6-ring PAHs, respectively. The 4-ring PAHs were predominant in WW1, WW3, and WW4 sludge samples, ranging from 40.7% to 41.7% (Figure 4); followed by 3- & 5-ring PAHs accounting for 21.0–24.1% and 19.1–24.0%, respectively. The 2- & 6-ring accounted for the lowest percentages of 5.4–9.2% and 6.0–9.7%, respectively. In the WW2 sludge sample, the percentage of 4-ring PAHs (47.6%) was also the highest. However, the percentage of other ring numbers PAHs to ΣPAHs is different from the other three types of sludge, which are 26.7% of 6-ring, 16.7% of 5-ring, 5.5% of 3-ring and 5.5% of 2-ring. Hua et al. [24] reported that the main components of PAHs in sewage sludge from 12 different industrial and economic development cities in Zhejiang Province (China) were 3- & 4-ring, accounting for 81–97%. Hu et al. [43] also reported that the main composition of PAHs in different types of sludge (including dying, beer-brewing, paper manufacturing, and municipal wastewater treatment plants containing domestic wastewater and industrial wastewater) is 4-ring (43–70%), followed by 3-ring (16–52%), which together accounted for 81–97% of ΣPAHs. Wołejko et al. [37] reported that the 4-ring and 3-ring PAHs in the sludge of the Sokółka WWTP in Poland accounted for 62% and 22%, respectively, accounting for 84% of the ΣPAHs. Overall, the PAHs of sewage sludge is dominated by 3- and 4-ring PAHs, of which 3-ring PAHs is most advantageous with phenanthrene, acenaphthylene, and fluorene, and 4-ring PAHs is most advantageous for fluoranthene, pyrene, and chrysene. However, some sewage sludge has also been found to be dominated by 5-ring benzo[b]fluoranthene, and benzo[a]pyrene or 6-ring benzo[g,h,i]perylene and DAB (Table 5).

## 4. Conclusions

An appropriate method for the analysis of 16 PAHs in DWTP and WWTP sludge by GC-MS was established. For individual PAHs, the recovery of this method ranged from 74.3 to 108.7% with a detection limit of 0.0010 to 0.0046 mg/kg dw. Using this method for the determination of the PAHs content of two DWTP and four WWTP sludge samples in southwestern Taiwan, the concentration of PAHs in WWTP sludge is between 0.5342–1.0666 mg/kg dw higher than 4–16 times DWTP sludge (0.0668–0.1357 mg/kg dw). These measured concentrations are lower than the PAHs limits applied to agricultural soils in the EU. The PAHs of the DWTP sludge samples in this study were mainly phenanthrene (22.7%) and anthracene (14.9%) of 3-ring PAH and pyrene (10.5%) and chrysene (10.9%) of 4-ring, and the PAHs of WWTP sludge were most advantageous with 4-ring fluoranthene (11.7%), pyrene (16.2%) and chrysene (10.3%). This result can be used for regular monitoring to establish a background for sludge PAHs to provide a reference for future sludge management and applied agriculture.

## Figures and Tables

**Table 1:** Parameters of the GC-MS system.

Parameter	Set Condition
Gas chromatography (GC)	Agilent 7890B (with Agilent 7693A autosampler)
Injection volume	1 µL
Inlet temperature	280 °C
Capillary column	HP-5MS (30 m × 0.25 mm i.d. with 0.25 μm film)
Injection mode	Splitless
Carrier gas	Helium, 1 mL/min
Temperature program	40 °C (1 min) → 120 °C (35 °C/min) → 160 °C (10 °C/min) → 300 °C (5 °C/min, hold for 10 min)
Mass selective detector (MS)	Agilent 5977A
Ionization mode	Electron ionization (EI)
Transfer line temperature	280 °C
Ion source temperature	230 °C
Quadrupole temperature	150 °C
Electronic energy	70 eV
Scan mode	Selective ion monitoring (SIM) (see Table 2)
Solvent delay	4 min

**Table 2:** Selected ion monitoring of each PAHs in GC-MS system.

Compounds	Abbreviation	Retention Time (min)	Selected Ions (m/z)
Naphthalene-d_8_	IS1 ^a^	4.679	**136** ^c^
Naphthalene	NA	4.964	**128**, 129, 127
2-Fluorobiphenyl	SS1	6.134	**172**
Acenaphthylene	ACY	6.845	**152**, 151, 153
Acenaphthene-d_10_	IS2	7.125	**164**
Acenaphthene	ACE	7.176	**154**, 153, 152
Fluorene	FL	8.177	**166**, 165, 167
Phenanthrene-d_10_	IS3	10.511	**188**
Phenanthrene	PH	10.557	**178**, 179, 176
Anthracene	AN	10.700	**178**, 176, 179
Fluoranthene	FLU	14.624	**202**, 229, 226
Pyrene	PY	15.399	**202**, 200, 203
4-Terphenyl-d_14_	SS2 ^b^	16.622	**244**
Benzo[a]anthracene	BaA	20.582	**228**, 229, 226
Chrysene-d_12_	IS4	20.626	**240**
Chrysene	CH	20.724	**228**, 226, 229
Benzo[b]fluoranthene	BbF	25.085	**252**, 253, 125
Benzo[k]fluoranthene	BkF	25.188	**252**, 253, 125
Benzo[a]pyrene	BaP	26.249	**252**, 253, 125
Perylene-d_12_	IS5	26.500	**264**
Indeno[1,2,3-cd]pyrene	IP	30.298	**276**, 138, 277
Dibenz[a,h]anthracene	DBA	30.571	**278**, 139, 279
Benzo[g,h,i]perylene	BP	31.026	**276**, 138, 277

^a^ Internal standard. ^b^ Surrogate standard. ^c^ Bold indicates quantitative ion.

**Table 3:** Response factor, detection limits, recoveries of check standards, and relative percent differences of sample duplicates for individual PAHs in this study.

PAHs ^a^	Response Factor (RF) (*n* = 5)		Check Standard (*n* = 3) R ^b^ (%)	Duplicate Sample (*n* = 6) RPD ^b^ (%)	Spike Sample (*n* = 3) P ^b^ (%)	Detection Limits (mg/kg dw)
	Average ± SD ^a^	RSD ^b^ (%)				
NA	1.11 ± 0.02	1.5	91 ± 3.5	6.1 ± 3.7	82.3 ± 0.6	0.0010
ACY	1.26 ± 0.04	3.3	94 ± 1.0	7.1 ± 4.6	108.0 ± 3.6	0.0012
ACE	1.17 ± 0.03	2.8	86 ± 0.6	3.4 ± 4.1	100.7 ± 3.1	0.0017
FL	0.95 ± 0.04	4.6	88 ± 1.2	7.1 ± 5.3	105.3 ± 5.5	0.0018
PH	1.18 ± 0.03	2.8	98 ± 0.3	5.5 ± 4.4	108.7 ± 2.9	0.0013
AN	0.64 ± 0.03	5.5	102 ± 2.1	8.1 ± 2.8	74.7 ± 2.5	0.0028
FLU	0.86 ± 0.05	5.6	93 ± 0.6	9.4 ± 3.6	76.0 ± 1.7	0.0022
PY	1.48 ± 0.11	7.4	98 ± 4.3	1.7 ± 0.9	76.3 ± 2.9	0.0045
BaA	0.42 ± 0.03	7.6	90 ± 3.3	4.8 ± 3.9	74.3 ± 2.3	0.0039
CH	1.20 ± 0.08	6.3	90 ± 2.4	7.1 ± 1.4	87.3 ± 2.1	0.0033
BbF	1.23 ± 0.05	4.2	96 ± 5.6	6.2 ± 3.7	94.3 ± 3.8	0.0032
BkF	1.36 ± 0.12	8.6	98 ± 2.8	7.5 ± 6.4	88.0 ± 0.0	0.0031
BaP	0.66 ± 0.05	8.3	92 ± 3.6	3.4 ± 1.5	93.3 ± 0.6	0.0045
IP	0.44 ± 0.04	9.4	98 ± 0.9	7.3 ± 2.4	90.3 ± 7.0	0.0046
DBA	0.59 ± 0.04	6.8	97 ± 8.1	8.5 ± 4.1	79.3 ± 1.5	0.0045
BP	1.81 ± 0.17	9.5	97 ± 2.9	5.5 ± 1.7	83.0 ± 1.0	0.0033
SS1	1.54 ± 0.11	7.4	102 ± 7.1	5.1± 2.7	92.3 ± 6.7	-
SS2	1.11 ± 0.02	1.5	107 ± 1.8	6.7± 3.7	89.5 ± 8.8	-

^a^ The definitions of compound abbreviation see Table 2; ^b^ SD: standard deviation; RSD: Relative standard deviation; R: Recoveries of check standard; RPD: Relative percent differences; P: Recoveries of spike sample.

**Table 4:** PAHs content (mg/kg dw) of sludge from selected drinking water treatment plants (DWTP) and wastewater treatment plants (WWTP) in southwestern Taiwan.

PAHs ^a^		DWTP		WWTP			
		DW 1	DW 2	WW 1	WW2	WW3	WW4
2-ring	NA	0.0117	ND (0.0008) ^e^	0.0981	0.0185	0.0290	0.0518
3-ring	ACY	ND (0.0007) ^e^	ND (0.0002) ^e^	0.0285	0.0009	0.0058	0.0146
	ACE	0.0019	0.0017	0.0064	0.0017	0.0028	0.0117
	FL	0.0054	0.0043	0.0104	0.0034	0.0139	0.0097
	PH	0.0201	0.0204	0.1291	0.0114	0.0511	0.1018
	AN	0.0396	ND (0.0005) ^e^	0.0827	0.0122	0.0384	0.0683
4-ring	FLU	0.0067	0.0075	0.1516	0.0129	0.0829	0.1275
	PY	0.0081	0.0100	0.1475	0.1336	0.0657	0.1195
	BaA ^b^	0.0119	ND (0.0014) ^e^	0.0568	0.0163	0.0236	0.0382
	CH ^b^	0.0107	0.0093	0.0884	0.0924	0.0452	0.0622
5-ring	BbF ^b^	0.0015	0.0042	0.0301	0.0087	0.0195	0.0257
	BkF ^b^	0.0018	ND (0.0006) ^e^	0.0297	0.0097	0.0186	0.0255
	BaP ^b^	0.0059	ND (0.0011) ^e^	0.0685	0.0070	0.0373	0.0527
	IP ^b^	0.0049	ND (0.0010) ^e^	0.0754	0.0640	0.0526	0.0750
6-ring	DBA ^b^	ND (0.0042) ^e^	ND (0.0002) ^e^	0.0228	0.0434	0.0134	0.0210
	BP	0.0008	0.0037	0.0408	0.0997	0.0344	0.0635
ΣPAHs ^c^		0.1357	0.0668	1.0666	0.5357	0.5342	0.8684
ΣLPAHs ^c^		0.0793	0.0279	0.0355	0.0480	0.1410	0.2577
ΣHPAHs ^c^		0.0564	0.0389	0.7116	0.4877	0.3931	0.6107
ΣLPAHs/ΣHPAHs		1.40	0.72	0.50	0.10	0.36	0.42
ΣCPAHs ^c^		0.0409	0.0177	0.3716	0.2415	0.2101	0.3002
ΣTEQ ^d^		0.0041	0.0018	0.0372	0.0242	0.0210	0.0300

^a^ The definitions of compound abbreviation see Table 2; ^b^ Carcinogenic PAHs; ^c^ ΣPAHs: sum of 2–6-ring PAHs; ΣLPAHs: sum of 2- & 3-ring PAHs; ΣHPAHs: sum of 4-, 5-, and 6-ring PAHs; ΣCPAHs: sum of 7 carcinogenic PAHs; ^d^ ΣTEQ: sum of 7 carcinogenic PAHs BaP toxic equivalence quotient; ^e^ The measured value is less than the detection limit.

**Table 5:** Compare the concentrations and composition of PAHs in sludge from other studies around the world.

Location	Sludge Type	ΣPAHs (mg/kg)	ΣCPAHs (mg/kg)	Dominant PAHs ^a^ (percentage)	Dominant PAHs Ring (percentage)	Ref.
China (Zhejiang)	Sewage (dom/ind) ^b^	33.73–82.58 56.7 ± 18.5	5.8–28.7 15.4 ± 7.5	PH (27) ^d^, FLU (12)	3 (42), 4 (30)	[24]
China (Beijing)	Sewage (dom/ind)	2.47–25.92 8.31 ± 8.79	2.08–23.0 6.15 ± 8.24	CH (10), BbF (22), BaP (15), BP (18)	4 (23), 5 (51), 6 (21)	[25]
China (Guangdong)	Sewage (dom/ind)	2.53–6.93 4.40 ± 2.27	0.70–1.01 0.87 ± 0.16	PH (30), FLU (16), PY (18)	3 (43), 4 (42)	[26]
China (Taian)	Sewage (dom/ind)	2.81–3.18 3.00 ± 0.26	0.12–0.61 0.36 ± 0.35	NA (26), PH (22), FLU (13)	2 (26), 3 (42), 4 (29)	[27]
China (Harbin)	Sewage (dom/ind)	2.2–20 8.2	Na ^c^	na	5&6 (55), 4 (25), 2 (20)	[28]
Japan	Sewage	0.069 ± 0.038	na	na	na	[18]
Turkey (Bursa)	Sewage (dom/ind)	1.78–19.9 6.08 ± 4.69	1.31–11.57 4.18 ± 2.77	na	na	[29]
Korea	Sewage (urban/rural)	1.30–44.9 10.4 ± 17.0	0.23–25.6 4.8 ± 10.2	FLU (14), PY (13), BbF (11)	4 (39), 5 (32)	[30]
Tunisia	Sewage (various)	0.096–7.72 1.25 ± 2.45	0.005–1.37 0.21 ± 0.44	PH (28), PY (16), NA (16)	3 (34), 4 (40), 2 (16)	[20]
Switzerland	Sewage (dom/ind/runoff)	1.01–22.6 7.10 ± 5.73	0.46–12.41 3.18 ± 3.25	PH (11), FLU (17), PY (14), BbF (11)	3 (17), 4 (44), 5 (29)	[31]
Kuwait	Sewage (urban)	2.01–7.76 4.33 ± 2.22	0.02–2.06 1.42 ± 0.74	PH (14), AN (11), DBA (11)	3 (45), 4 (23), 5 (19)	[32]
Italy (Venice)	Sewage (urban)	1.26–1.44 1.35 ± 0.13	0.57–0.73 0.65 ± 0.11	PY (8.7), BaA (8.6), CH (8.2)	3 (28), 4 (32), 5 (26)	[19]
Spain (Catalonia)	Sewage (urban)	1.13–5.52 3.02 ± 1.55	0.34–2.25 0.76 ± 0.64	PH (25), PY (13), FLU (9.0)	3 (43), 4 (31)	[33]
Spain (Cadiz)	Sewage (urban)	1.97–10.1 4.97 ± 1.9	0.47–4.61 1.93 ± 0.99	ACY (11), PH (9.3), PY (19)	3 (28), 4 (38)	[34]
India (Delhi)	Sewage	14.9–24.2 20.67 ± 4.14	9.81 ± 2.35	BP, DBA.	6 (33), 5 (31)	[35]
Poland	Sewage	2.04–36.44 11.61 ± 8.72	4.30	ACY (18), FLU (17), BbF (16)	3 (34), 4 (39)	[36]
Poland	Dairy sewage	0.498	0.12	ACY (2), FL (13), PY (21)	3 (45), 4 (36)	[16]
United Kingdom	Sewage	18–94 44.8	4.5–27.6 13.2	FL (13), PH (17), FLU (11)	3 (39), 4 (30)	[1]
Jordan (Karak)	Sewage (dom/ind)	0.029–0.039 0.034 ± 0.005	0.009–0.016 0.013 ± 0.004	FL (14), PH (17), BP (17)	3 (34), 4 (30), 6 (21)	[17]
Taiwan	Sewage (urban)	0.53–1.07 0.75 ± 0.26	0.021–0.037 0.028 ± 0.007	PY (16), FLU (12), CH (10)	3 (19), 4 (42), 5 (20)	This study

^a^ The definitions of compound abbreviation see Table 2; ^b^ dom: domestic, ind: industrial; ^c^ Not available; ^d^ The values in parentheses indicate the percentage to total PAHs.
